# An antitumor peptide RS17‐targeted CD47, design, synthesis, and antitumor activity

**DOI:** 10.1002/cam4.3768

**Published:** 2021-02-24

**Authors:** Xinmin Wang, Ying Wang, Jialiang Hu, Hanmei Xu

**Affiliations:** ^1^ The Engineering Research Centre of Peptide Drug Discovery and Development China Pharmaceutical University Nanjing Jiangsu China; ^2^ State Key Laboratory of Natural Medicines Ministry of Education China Pharmaceutical University Nanjing China

**Keywords:** antitumor drug, CD47, peptide, SIRPα, targeted therapy

## Abstract

**Background:**

CD47 is a widely expressed transmembrane protein located on the surface of somatic cells. It mediates a variety of cellular processes including apoptosis, proliferation, adhesion, and migration. An important role for CD47 is the transmission of a “Don't eat me” signal by interacting with SIRPα on the macrophage surface membrane, thereby preventing the phagocytosis of normal cells. However, cancer cells can take advantage of this autogenous signal to protect themselves from phagocytosis, thus enabling immune escape. Blocking the interaction between CD47 and SIRPα has proven to be effective in removing cancer cells. The treatment of various cancers with CD47 monoclonal antibodies has also been validated.

**Methods:**

We designed and synthesized a peptide (RS17), which can specifically bind to CD47 and block CD47‐SIRPα signaling. The affinity of RS17 for CD47‐expressing tumor cells was determined, while the inhibition of CD47‐SIRPα signaling was evaluated in vitro and in vivo.

**Results:**

The results indicated that RS17 significantly promotes the phagocytosis of tumor cells by macrophages and had a similar therapeutic effect compared with a positive control (CD47 monoclonal antibodies). In addition, a cancer xenograft mouse model was established using CD47‐expressing HepG2 cells to evaluate the effect of RS17 on tumor growth in vivo. Using ex vivo and in vivo mouse models, RS17 demonstrated a high inhibitory effect on tumor growth.

**Conclusions:**

Based on our results, RS17 may represent a novel therapeutic peptide for cancer therapy.

## Graphical abstract

A antitumor peptide RS17 targeted CD47, Design, Synthesis and Anti‐Tumor activity.

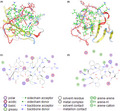

## 1 INTRODUCTION

CD47 (also called integrin associated protein) is a ubiquitously expressed membrane protein encoded by the human *CD47* gene.^1^ It belongs to the immunoglobulin superfamily with an uncommon structure.^2^ CD47 is a glycoprotein of approximately 52 kDa that consists of a short C‐terminal intracellular tail, a five‐transmembrane‐domain and an N‐terminal IgV extracellular domain. There are typically four alternatively spliced cytoplasmic C‐terminal forms of CD47 in vivo, with Form‐2 representing the most abundantly expressed transcript.^2^, ^3^ The cytoplasmic tails lack a significant signaling domain and the function of the cytoplasmic tail remains unknown.^2^, ^4^

SIRPα and TSP‐1 are two high‐affinity CD47 ligands.^5^ The interaction of CD47 with its ligands affects a variety of cell processes. Thus, CD47 plays an important role in the process of inflammation and angiogenesis.^4^, ^6^ In addition, CD47 also interacts with some typical transmembrane integrins including the well‐characterized integrin αVβ3.^7^ The interactions of these integrins with CD47 attenuate cell functions including spreading, migration, and adhesion.^1^, ^7^ However, recent studies of CD47 function have mainly focused on the CD47–SIRPα interaction which inhibits phagocytosis.^8^ SIRPα has many aliases including BIT, SHPS‐1, and CD172a. It is an administrative transmembrane glycoprotein belonging to the SIRP family and expressed primarily by macrophages, dendritic cells, neurons, and stem cells.^9^, ^10^ SIRPα consistently behaves as a negative receptor and interacts with CD47 to generate the anti‐phagocytic signal, which negatively regulates the function of innate immune cells such as immune homeostasis.^10^, ^11^ The corresponding intracellular event is the generation and accumulation of myosin IIA which finally inhibit the process of phagocytosis.^12^, ^13^

CD47 were expressed in a variety of human tumors such as non‐Hodgkin's lymphoma, bladder cancer, breast cancer, and acute myeloid leukemia.^14^, ^15^, ^16^, ^17^ Although CD47 has some impact on the proliferation and migration of tumor cells,^18^, ^19^ it functions in cancer cells as a cell surface ligand. Through interactions with SIRPα on surrounding phagocytes, it generates an antiphagocytic signal to macrophages.^10^, ^13^ Overexpression of CD47 enables cancer cells to escape phagocytosis. Therefore, CD47 is a potential drug target for cancer immunotherapy and anti‐CD47 antibodies were found to effectively release the antiphagocytic signal for macrophages to clear CD47‐expressing tumor cells.^20^, ^21^

Peptides are unique pharmaceutical compounds with many favorable properties including excellent target selectivity, low toxicity, and outstanding efficacy.^22^ Some peptides are actively involved in various physiological mechanisms and behave as growth factors, neurotransmitters, antimicrobials, and hormones.^23^, ^24^, ^25^, ^26^ Peptides can be lead compounds in drug development. Their highly specificity in target binding, selectivity for target molecules, flexibility in amino acid sequences, and potential binding renders peptides excellent drug candidates.^27^, ^28^ Compared to large biomolecules, peptides can penetrate deeper into tissues. In addition, compared to antibodies and recombinant proteins, peptides are less immunogenic, more potent, minimally toxic, relatively inexpensive, easy to manufacture and store.^27^, ^28^, ^29^, ^30^, ^31^, ^32^, ^33^

In the past decade, targeted therapies have become an important way of cancer treatment.^34^ Many targeted drugs including those in clinical trials or in the clinic use inhibit tumor growth by regulating tumorigenesis, angiogenesis, and progression.^35^ Recently, peptide drugs became a promising class of drug candidates. They composed one of the largest areas of drug development, especially in oncology as well as metabolic and cardiovascular diseases.^22^ CD47 antagonist peptide is a primary example of anticancer peptide drug and CD47 became a potential anticancer target as a novel treatment.

In this work, a peptide RS17 with novel sequence was designed and synthesized that targets the CD47‐SIRPα signaling pathway.^36^ To evaluate the RS17 peptide and CD47 interaction, an Molecular Operating Environment (MOE 2009) software was used for docking simulation analysis.^37^ We validated the RS17/CD47 interaction by microscale thermophoresis (MST) and flow cytometry analysis. Furthermore, the antitumor activity of RS17 was evaluated in vivo and in vitro.

## 2 MATERIALS AND METHODS

### 2.1 Design and virtual screening of RS17 and its interaction with CD47

MOE 2009 software was from Chemical Competing Group, Quebec, Canada. We used this software to analyze CD47 and SIRPα interactions (PDB ID: 2jjs) and identified six residues in CD47 (Leu3, Lys6, Tyr37, Glu97, Glu104, and Glu106) which may contribute to the interaction. Using these six amino acids, we designed the CD47 targeting peptide, RS17, by homology modeling based on SIRPα and CD47 interaction. The complex structure of the interaction between CD47 and SIRPα was also considered in the design process. Then, MOE 2009 software was used to determine the docking image between RS17 and CD47.

### 2.2 Synthesis and detection of RS17

The peptides were synthesized using the solid‐phase peptide synthesis method. Amino acid activation phase included 1‐hydroxybenzotriazole (HOBt) and N, N′‐diisopropylcarbodiimide (DIC). Deprotection agent was piperidine (PIP). Following synthesis, the peptides were cleaved with 90% of trifluoroacetic acid (TFA) and precipitated with ether to obtain the crude product. The crude peptides were purified by HPLC and the molecular weight were measured by online ESI‐MS, respectively. Besides, the FITC‐RS17 peptide was prepared by coupling with FITC through the Acp active group to RS17. The product was purified and analyzed by the same process.

### 2.3 Cell culture

The SCC‐13 human cutaneous squamous cell carcinoma cell lines, HepG2 human HCC cell line, THP‐1 human mononuclear cell line, and mouse mononuclear/macrophage cell lines were from Chinese Academy of Sciences in Shanghai. SCC‐13 and HepG2 cells were incubated in DMEM medium which contains 100 μg/ml of streptomycin and 50 μg/ml of penicillin. The medium also contains 10% of fetal bovine serum from Gibco Life Technologies. RAW264.7 cells were cultured in RPMI 1640 medium. As the DMEM medium, the RPMI 1640 medium also contains 100 μg/ml of streptomycin, 50 μg/ml of penicillin, and 10% of FBS. THP‐1 cells were cultured in RPMI 1640 medium which contains 100 μg/ml of streptomycin, 50 μg/ml of penicillin, 2200 μg/ml of NaHCO_3_, 0.05 mM of β‐mercaptoethanol, and 10% of FBS. All cells were cultured in a humidified incubator at 37°C in 5% CO_2_.

### 2.4 Western blot analysis

Cellular protein expression was measured by western blot analysis.^38^ Tumor cells and macrophages were enzymatically collected and after centrifugation they were washed with PBS precooled at 4℃. Cell lysis was done using RIPA lysis buffer placed in ice for 30 min (Wanleibio). The undissolved cell fragments were removed by centrifugation at 12000 *g* at 4℃ for 5 min. Proteins in the supernatant were developed on 10% of SDS‐PAGEs and after electrophoresis the protein bands on the gels were transported to PVDF membranes. After blocking the PVDF membranes with 5% of skim milk at room temperature for 2 h, the membrane was incubated separately with first antibodies (Abcam) specific for target proteins overnight at 4℃. After briefly washing the PVDF membranes with TBST for three times, the membranes were incubated with horseradish peroxidase‐conjugated second antibody (Wuhan Servicebio) at room temperature for 1.5 h. After washing the membranes with TBST buffer, bands for target proteins were visualized with an ECL detection kit (Millipore Corporation).

### 2.5 Microscale thermophoresis analysis

The technique of Microscale Thermophoresis (MST) is widely used in determining the affinity of the interaction between biomolecules based on thermal motion.^39^ Hence, MST can describe and predict the binding between proteins and peptides. CD47 protein was from Cloud‐Clone. The CD47 protein was dissolved in a buffer solution without primary amine compounds and exchanged by mixed column A. The CD47 protein was stained by Cy5 fluorescent dye (Ruixin Biological Technology) and after staining, the labeled protein was purified by column B. Finally, the RS17 peptides were diluted and mixed with the labeled protein in a 1:1 ratio. After incubating at room temperature for 5 min, samples were detected and data were processed by Monolith NT.115 and NT‐Analysis, which were from NanoTemper.

### 2.6 Flow cytometry analysis

The binding of RS17 to the CD47‐expressing cells were assessed by the Flow cytometry technique.^14^ In brief, HepG2 and SCC‐13.^14^ In brief, HepG2 and SCC‐13 cells were collected and adjusted to 3 × 10^6^ cells/ml. A 100 μl cell suspension per tube was incubated with RS17 in a humidified environment for 1 h (5% CO_2_, 37℃, in dark). After washing with PBS, the cells were analyzed with a flow cytometer (Miltenyi Biotec GmbH). Data were analyzed with FlowJo 7.6.

### 2.7 Macrophage polarization

THP‐1 cells were cultured normally until logarithmic phase when phorbol 12‐myristate 13‐acetate (PMA, Beyotime) was added at a final concentration of 100 ng/ml. The original medium was replaced with a non‐PMA RPMI 1640 medium after a 48‐h incubation. The cells were cultured for an additional 24 h until THP‐1 cells were transformed into M0 phenotype macrophages. LPS (1 μg/ml) (Beyotime) was added to the medium to transform M0 phenotype macrophages into M1‐polarized macrophages after 48 h.^40^ RAW264.7 cells were cultured normally until logarithmic phase. LPS (1 μg/ml) was added to transform M0 phenotype macrophages into M1‐polarized macrophages after 48 h.^41^

### 2.8 In vitro phagocytosis

5 × 10^4^ polarized macrophages in RPMI 1640 medium were seeded into each well of a 24‐well plate in presence of 10% FBS. After 24 h, the cells were incubated with serum‐free medium. After incubating for 2 h, CFSE (Invitrogen)‐tagged HepG2 cells were added to the plates at 2 × 10^5^ cells/well. Then, 1 μM B6H12 (BioXcell) or RS17 were added to the 24‐well tissue culture plate. The original medium was replaced with PBS after 2 h and the cells in the plate were observed under an OLYMPUS IX53 fluorescence microscope. To monitor green fluorescence, the excitation wavelength was set to 490–495 nm.^14^ The number of CFSE‐positive cells within macrophages was counted and the phagocytic index was determined as the number of ingested cells per 100 macrophages. At least 200 macrophages were counted per well.^42^

Prior to flow cytometry, the co‐incubated macrophages were collected and incubated with APC‐Anti‐CD11b antibodies (BioLegend) in a humidified environment for 1 h (5% CO_2_, 37℃, without light). After collection and washing twice with PBS, the cells were measured using a flow cytometer (Miltenyi Biotec GmbH). FlowJo 7.6 data analysis was done to screen CFSE^+^ CD11b^+^ cells.^42^

### 2.9 Lentiviral transfection

RFP (CMV‐Puro) lentiviral particles were purchased from HanBio. 5 × 10^4^ HepG2 in DMEM medium were seeded into each well of a 12‐well plate in presence of 10% FBS. A 6 μg/ml of Polybrene and 20 MOI lentivirus were added to each well. The cells were resuspended in DMEM medium in presence of 8 μg/ml puromycin after a 48‐h transfection. DMEM medium with 8 μg/ml of puromycin was replaced every 2 days for 1 week. RFP‐positive HepG2 cells were then acquired by flow cytometry. Polybrene and puromycin were from Sigma.

### 2.10 Antitumor activity by ex vivo assay

Female Balb/c nude mice (4–5 weeks) were bred in the absence of pathogens to assess the antitumor activity of indirect therapies in vivo.

RFP‐positive HepG2 cells at a concentration of 2 × 10^7^/ml were first co‐incubated for 1 h with 1 μM B6H12 (positive control) or RS17. The nude mice were separated into three groups (5 per group) and subcutaneously injected with 200 μl RFP‐positive HepG2 cells into the ventral side. Normal feeding lasted for 7 days. Body weight and tumor volume were measured in nude mice. Tumor dimensions were measured in two directions with and the formula of ½ × *a* × *b* ^2^ (*a*, length; *b*, width) was used to calculate tumor volume. The intravital fluorescence intensity of the tumors were detected with PerkinElmer IVIS Spectrum on day 7.

### 2.11 In vivo antitumor activity assay

Nude mice were subcutaneously injected with 2 × 10^7^ RFP HepG2 cells into the ventral side and separated into three groups (5 per group). One of the groups was selected as the experimental group and received a daily subcutaneous injection with RS17 (20 mg/kg), the other two groups represented a positive control and a blank control and received a daily subcutaneous injection with B6H12 (20 mg/kg) and PBS, respectively. Daily injections were administered for 28 days. Body weight, tumor volume, and imaging on day 28 were carried out described above.

## 3 RESULTS

### 3.1 Design and virtual screening of RS17 and their interactions with CD47

**FIGURE 1:**
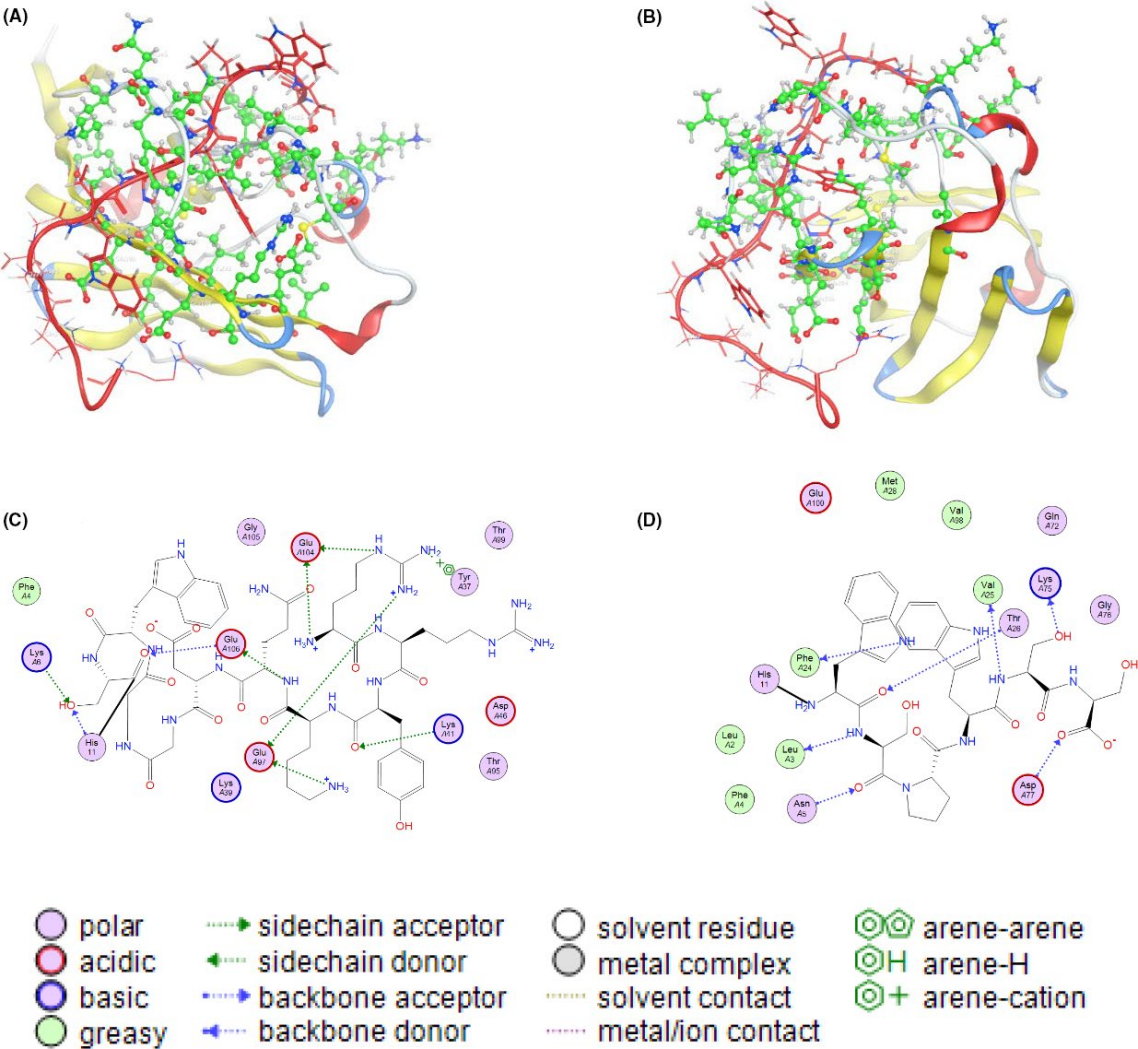
Interaction between RS17 and CD47. (A, B) Schematic diagram of the interaction between RS17 (red) and CD47 (green) from different perspectives. (C) The interaction between the N‐terminus of RS17 and CD47. (D) The interaction between the C‐terminus of RS17 and CD47

Based on the X‐ray diffraction images of CD47 and SIRPα crystal structure (PDB ID: 2jjs), we identified six candidate amino acid residues (Leu3, Lys6, Tyr37, Glu97, Glu104, and Glu106) in CD47 which may play a role in binding. We then designed a peptide, RS17 (RRYKQDGGWSHWSPWSS), predicted to bind to the key amino acids in CD47 and block SIRPα and CD47 interaction. We simulated the interaction between CD47 and RS17 using MOE 2009 software. The schematic diagram of the interaction between CD47 and RS17 is shown in Figure 1. RS17N‐terminus (RRYKQDGGWS) can interact with six amino acid residues (Lys6, Tyr37, Lys41, Glu97, Glu104, and Glu106) of CD47, while the C‐terminus (HWSPWSS) interacts with seven other amino acid residues (Leu3, Asn5, Phe24, Val25, Thr26, Lys75, and Asp77) in CD47.

### 3.2 Synthesis and detection of RS17

**FIGURE 2:**
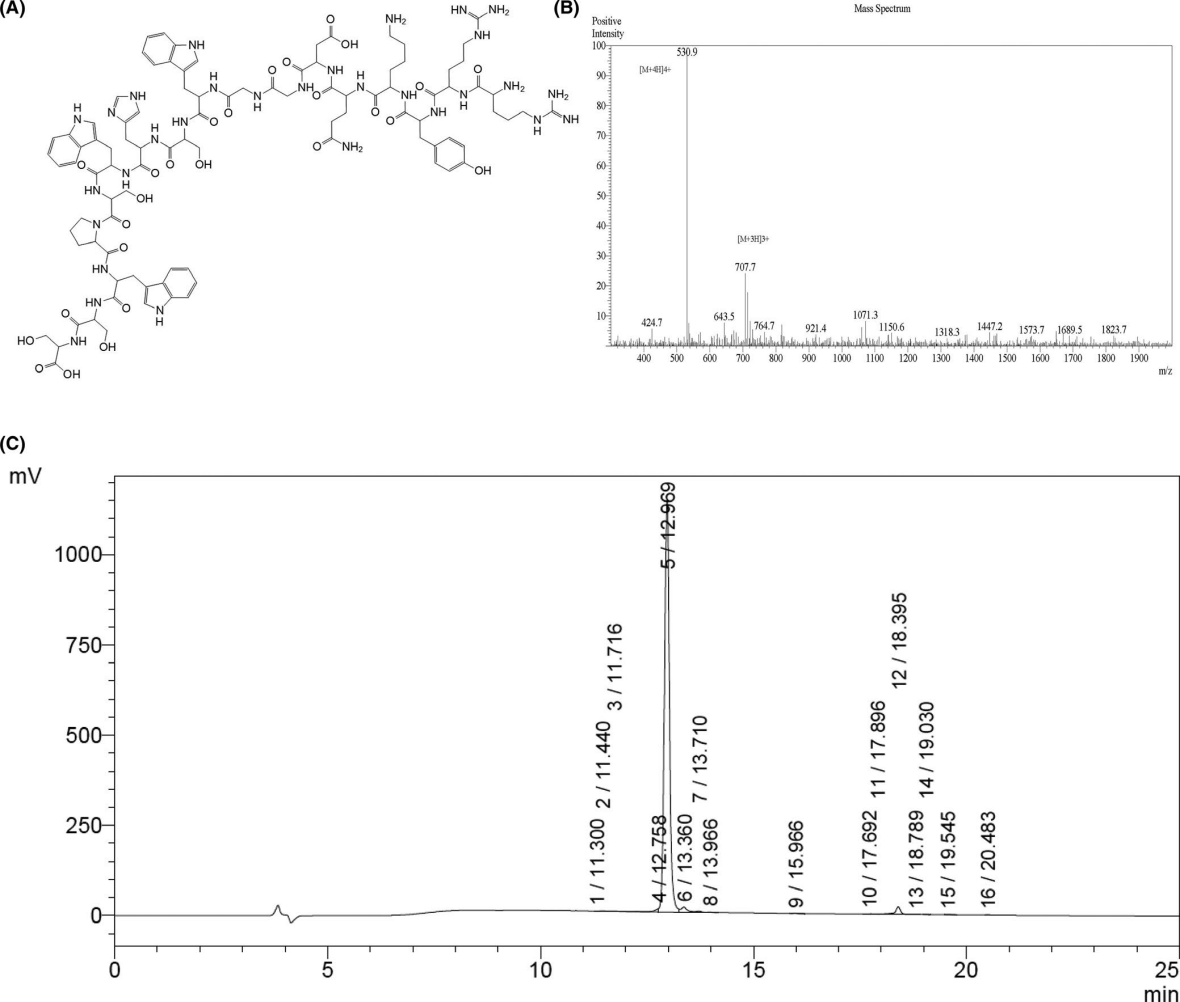
Chemical structure, molecular weight, and purity of the RS17 peptide. (A) Chemical structure of RS17. (B) Electrospray ionization mass spectrometry (ESI‐MS) indicating a molecular weight of 2119.6. (C) The purity of RS17 was 95.3% as determined by high performance liquid chromatography (HPLC)

The peptides used in the experiments were generated by solid‐phase synthesis. After synthesizing the RS17 peptide (Figure 2A), we analyzed its purity by HPLC and determined its molecular weight by mass spectrometry (MS). The RS17 molecular weight was measured to be 2119.6 which is consistent with the theoretical value and its purity was 95.3% (Figure 2).

### 3.3 Expression of CD47 and SIRPα

**FIGURE 3:**
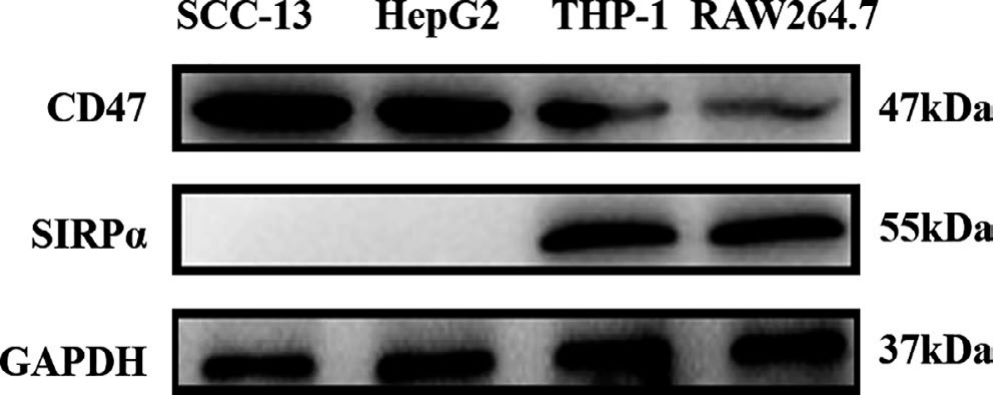
CD47 and SIRPα expression was measured in cell lines by western blot analysis. CD47 was detected in all of four cell lines, whereas SIRPα was only detected in the mononuclear/macrophage cell lines

The engagement of tumor cell CD47 with SIRPα expressed on the surface of monocytes/macrophages may produce inhibitory signals which inhibit phagocytosis.^10^ Because the effect of CD47 antagonists for cancer treatment requires blocking CD47 and SIRPα interaction, we examined CD47 expression on selected cells. An experimental model was established for evaluation of RS17 activity in vitro. The SCC‐13 cutaneous squamous cell carcinoma cell line and HepG2 hepatoma cell line exhibited high CD47 expression. In addition, we also screened mononuclear and macrophages for SIRPα expression and found that the THP‐1 human mononuclear and the RAW264.7 mouse mononuclear/macrophage cell lines expressed high SIRPα levels. As shown in Figure 3, high CD47 expression in both SIRPα‐expressing cells indicates that CD47 is a widely expressed cell surface factor.^11^

### 3.4 Affinity of RS17 with its target

**FIGURE 4:**
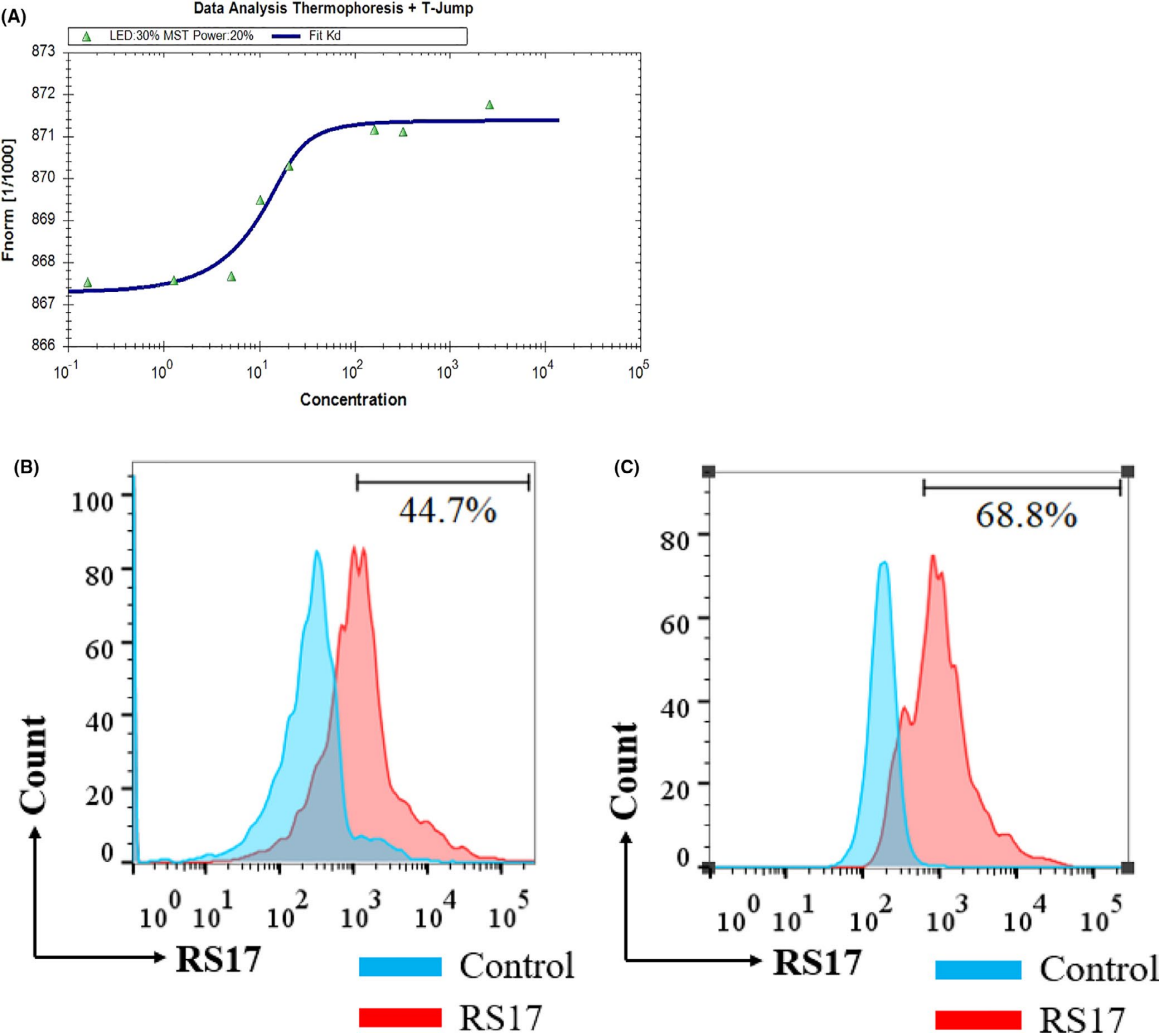
The interaction between RS17 and CD47. (A) Analysis of the data from the MST of RS17 and CD47. There was a high affinity between RS17 and CD47 (Kd = 3.857 ± 0.789 nM). (B) The interaction of FITC‐RS17 polypeptide and SCC‐13 cells as detected by flow cytometry after incubation of SCC‐13 cells with 1 μM FITC‐RS17. (C) The interaction of FITC‐RS17 polypeptide and HepG2 cells as detected by flow cytometry after incubation of HepG2 cells with 1 μM FITC‐RS17

Microscale thermophoresis (MST) is an effective technique that accurately quantitates the interaction between two molecules. It depends on the thermo‐swimming movement of a molecule, which is a directional transfer of the molecule across a temperature gradient dependent on molecular properties such as molecular size, electric charge, and conformation.^39^ A Monolith NT.115 instrument was used as a platform to perform the MST experiment and the results were analyzed using the accompanying NT‐Analysis software. As shown in Figure 4A, MST measurements revealed that the dissociation constant (Kd) for RS17 and CD47 interaction was 3.857 ± 0.789 nM indicating that there is high‐affinity binding between RS17 and CD47.

Moreover, we also used flow cytometry to assess binding of FITC‐RS17 with SCC‐13 and HepG2 cells, which highly express CD47. As shown in Figure 4B,C, FITC‐RS17 was effectively bound when co‐incubated with 1 μM FITC‐RS17 peptide. The results indicated that RS17 can not only bind to the CD47 molecule efficiently, but also shows a possibility which binds to cells exhibiting CD47 expression.

### 3.5 RS17 disrupts CD47–SIRPα interactions and promotes the phagocytic activity of macrophages

**FIGURE 5:**
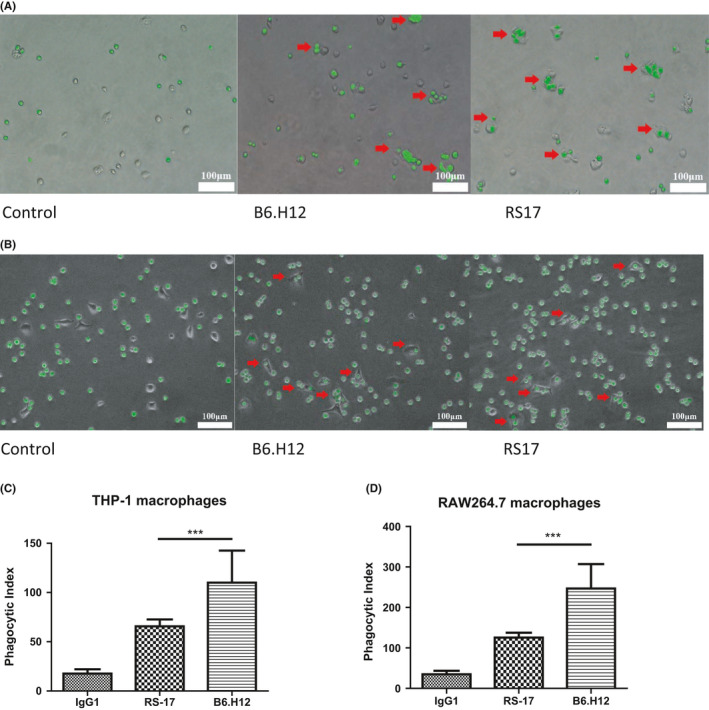
RS17 induced tumor cell phagocytosis by macrophages. (A) THP‐1 macrophages (M1) were co‐incubated with HepG2 cells. HepG2 cells were stained with the CFSE green dye and B6H12 was used as a positive control. (B) RAW264.7 macrophages (M1) were co‐incubated with HepG2 cells. HepG2 cells were stained with CFSE green dye and B6H12 was used as a positive control. (C) The phagocytic index of THP‐1 cells co‐incubated with HepG2 cells. The computational method is described in the “Materials and Methods” section. (D) Phagocytic index of RAW264.7 cells co‐incubated with HepG2 cells. Histogram results are presented as the mean ±SD (*n* = 3), **p < 0.01, ***p < 0.001

CD47 expressed on the tumor cells serves as a ligand for the SIRPα immune inhibitory receptor, which is expressed on macrophages. CD47 provides an antiphagocytic signal to macrophages by interacting with SIRPα. The idea that CD47 antibodies, such as B6H12, can prevent this interaction, and thus, enable macrophages to phagocytize CD47‐expressing tumor cells suggests that the RS17 peptide can produce the same effect.^11^ According to the method of Chao et al.,^14^ we used a phagocytosis experiment to verify that RS17 can promote the phagocytosis of CD47‐expressing HepG2 tumor cells by macrophages. Human THP‐1 monocytes and mouse RAW264.7 monocytes/macrophages were polarized to the M1 stage for this experiment. As expected, RS17 significantly increased phagocytosis of Carboxyfluorescein succinimidyl ester (CFSE)‐labeled HepG2 cells by macrophages. Incubation with both RS17 and CD47 antibodies (B6H12) resulted in increased phagocytosis by macrophages, whereas both macrophages weakly phagocytosed HepG2 cells in the absence of RS17 or B6H12 (Figure 5).

For a more accurate analysis of phagocytosis, we also used flow cytometry to quantify the effect of phagocytosis induced by RS17. CFSE‐labeled HepG2 cells, which were co‐incubated with THP‐1/RAW264.7(M1), were analyzed by flow cytometry. When macrophages phagocytosed CFSE‐labeled HepG2 cells, green fluorescence was detectable in the cells.^42^ The flow cytometry detection confirmed that RS17 significantly improved the efficiency of phagocytosis, and both RS17 and CD47 antibodies (B6H12) enhanced the phagocytic efficiency of macrophages (Figure S1).

### 3.6 RS17 inhibits the growth of high CD47‐expressing tumors of in vivo

Previous experiments identified the antitumor effect of RS17 in vitro. RS17 can inhibit tumor immune escape by blocking the CD47‐SIRPα signaling pathway. To determine the activity of RS17 in vivo, we designed two experiments to verify the antitumor activity of RS17 in vivo based on the method of Chao et al.^14^

**FIGURE 6:**
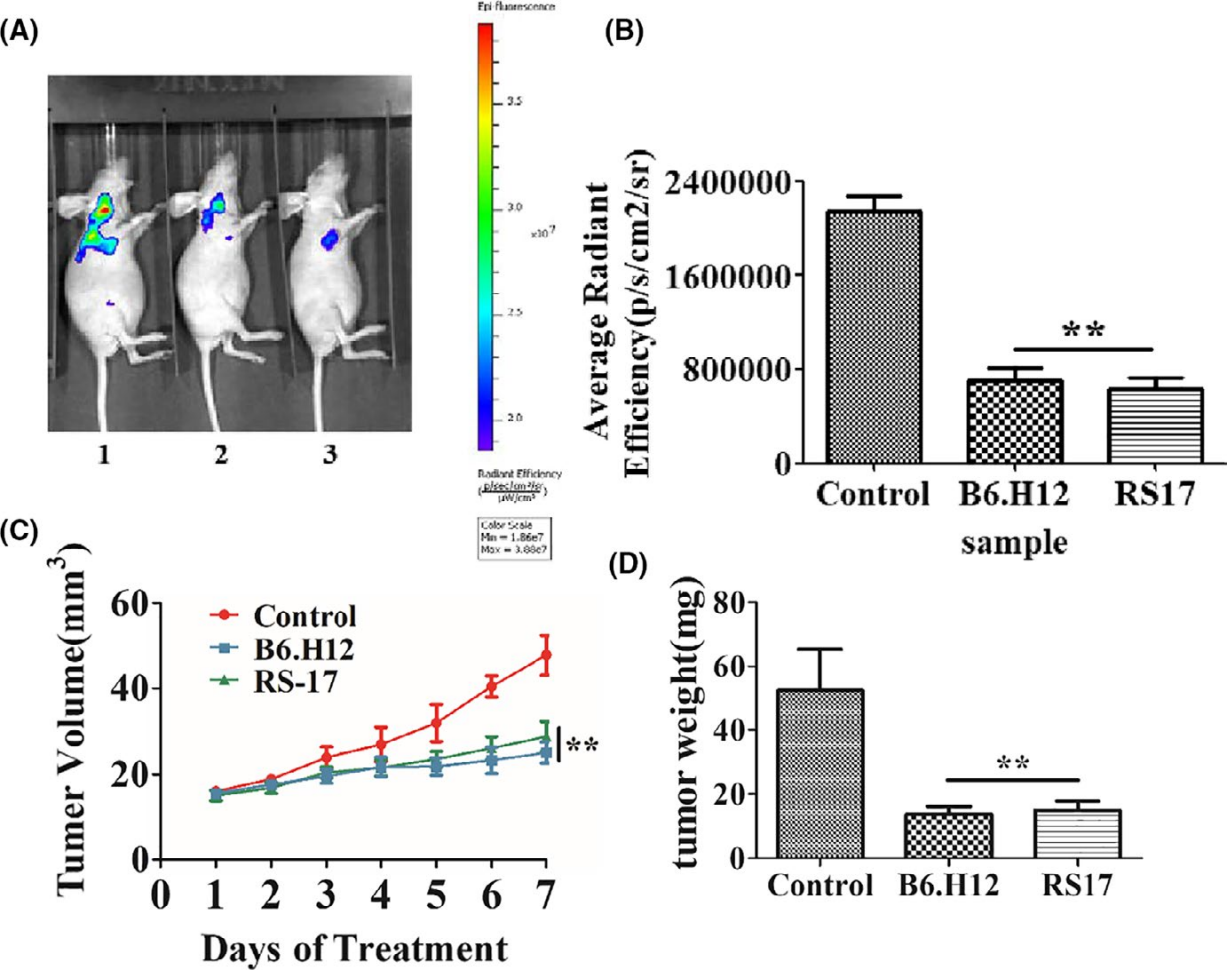
RS17 inhibits tumor growth in an ex vivo experiment. Nude mice were fed normally after implantation of tumor cells until day 7. (A) Analysis by in vivo imaging on day 7 (1‐PBS control, 2‐B6H12 positive control, 3‐RS17). (B) In vivo imaging results were quantified by average radiant efficiency. (C) The changes in tumor volume in nude mice. (D) Tumor weight in nude mice on day 7. Results are presented as the mean ± SD (*n* = 5), *p < 0.01, ** *p* < 0.001

First, an ex vivo experiment was performed. After pretreating and co‐incubating with the B6H12 antibody or RS17, RFP‐positive HepG2 cells, derived by transfection with lentivirus (RFP‐puromycin), were injected subcutaneously into nude mice. The mice were separated into three groups according to the treatment protocol. Body weight and tumor volume were measured during the feeding period. Seven days after normal feeding, all of the nude mice were examined by in vivo imaging. As shown in Figure 6, compared with the phosphate buffer saline (PBS) group, fluorescence in nude mice inoculated with RS17 peptide or B6H12 phagocytized RFP‐positive HepG2 cells significantly less. This result indicated that RS17 peptide inhibited tumor growth in vivo. The therapeutic effect of RS17 compared with anti‐CD47 antibodies. In addition, the experimental nude mice did not show a significant weight loss and the two treatments were well tolerated.

**FIGURE 7:**
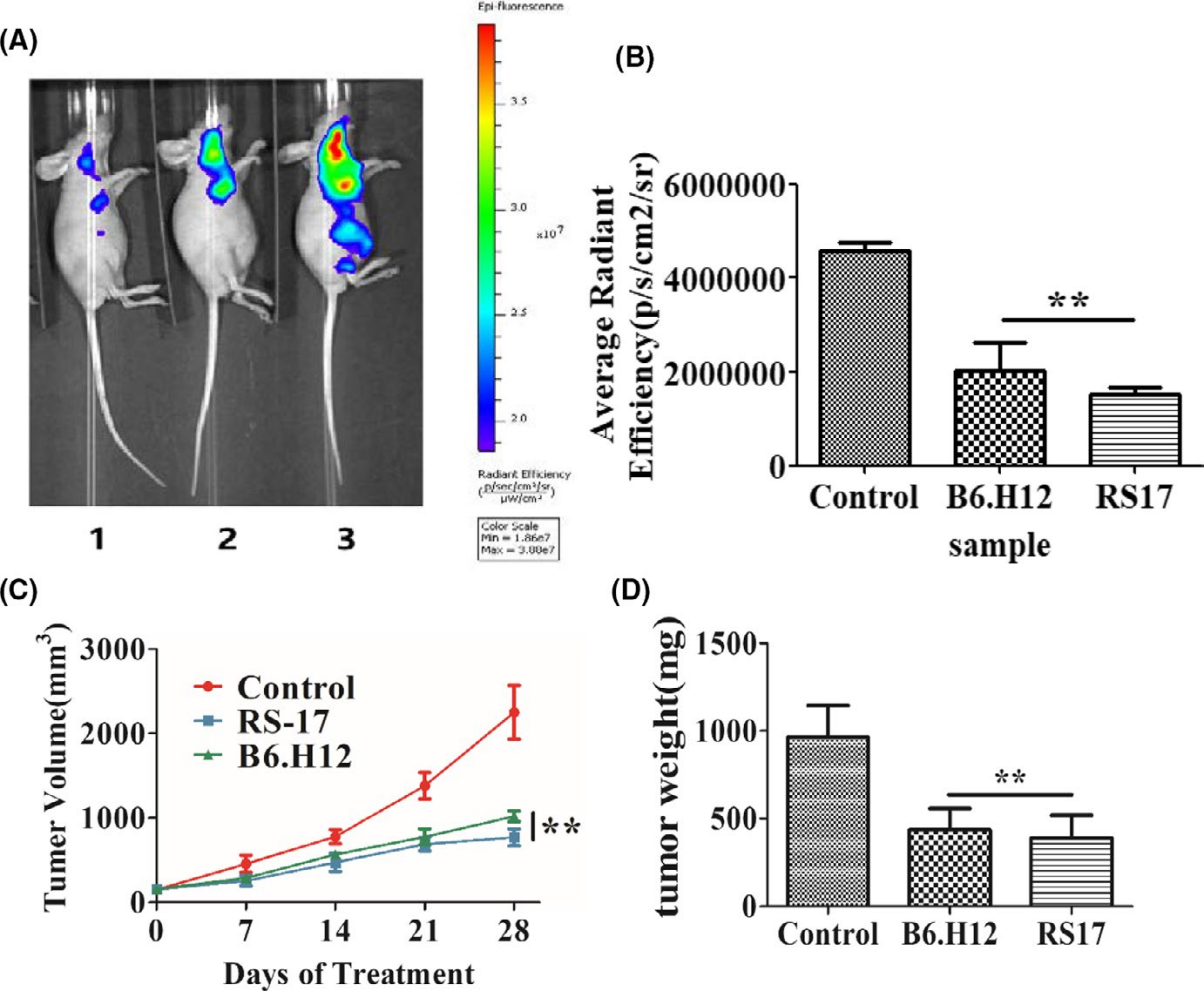
RS17 inhibition of tumor growth in vivo. Nude mice were subcutaneously implanted with tumor cells and treated with a subcutaneous injection of RS17 (20 mg/kg), B6H12 (20 mg/kg), or PBS once a day for 28 days. (A) Analysis by in vivo imaging on day 28 (1‐mouse from the RS17 treatment group, 2‐mouse from the B6H12 treatment group, 3‐mouse from the PBS control group). (B) In vivo imaging results were quantified by the average radiant efficiency. (C) The changes tumor volume in mice. (D) Tumor weight in mice on day 28. The results are presented as mean ± SD (*n* = 5), *p < 0.01, ** *p* < 0.001

An in vivo experiment was done to verify the therapeutic efficacy of RS17. First, RFP‐positive HepG2 cells were transfected with lentivirus (RFP‐puromycin) and subcutaneously injected into nude mice. The mice were separated into three groups and injected subcutaneously with RS17 (20 mg/kg), B6H12 (20 mg/kg), or PBS once a day for 4 weeks. Body weight and tumor volume were measured and all mice were subjected to in vivo imaging system at week 4. As shown in Figure 7, the growth of the tumors treated with RS17 or B6H12 was significantly inhibited. Moreover, RS17 polypeptide inhibited tumor growth in vivo, and the treatment effect of RS17 superior compared with that of the B6H12 anti‐CD47 antibody.

## 4 DISCUSSION

In this study, we used a bioinformatics method to design a CD47‐targeted peptide (RS17). MOE analysis confirmed that RS17 binds specifically to the extracellular domain of CD47. A solid‐phase peptide synthesis method was used to generate RS17 at 95.3% purity. MST and flow cytometry analysis confirmed that RS17 binds with high affinity to CD47. Functionally, RS17 incubation promoted phagocytosis of HepG2 human hepatocellular carcinoma cells by macrophages in vitro as determined by microscopy and flow cytometry. Furthermore, blocking the SIRPα/CD47 interaction with RS17 significantly inhibited the growth of HepG2 cells in Balb/c nude mice.

The RS17 peptide interacted with human CD47 on two human tumor cell lines (Figure 4). RS17 incubation increased phagocytosis of HepG2 cells by not only THP‐1 cells, but also RAW264.7 cells as determined by fluorescence microscopy. This result was further confirmed by detecting the phagocytosis of HepG2 cells by THP‐1 cells (Figure S1A) and RAW264.7 cells (Figure S1B) using flow cytometry. A positive antihuman CD47 control antibody (B6H12) exhibited a similar effect as RS17, which confirmed that the enhanced effect of RS17 on HepG2 phagocytosis occurred by inhibiting the CD47 and SIRPα interaction. The results in Figure 5 and Figure S1 show that human CD47 can interact with SIRPα on both THP‐1 and RAW264.7 cells. This is consistent with the in vivo tumor growth inhibition shown in Figures 6 and 7, in which RS17 or B6H12 treatment inhibited HepG2 growth by blocking the interactions between CD47 on HepG2 and SIRPα on mouse macrophages.

In vitro phagocytosis experiments (Figure 5; Figure S1) revealed that the effect of the B6H12 antibody was significantly higher compared with RS17, whereas in the in vivo tumor growth inhibition experiments (Figures 6 and 7), RS17 showed a significantly stronger inhibitory effect compared with B6H12. Both reagents were subcutaneously injected and RS17 was better absorbed compared with the B6H12 antibody via this route of administration. As a relatively small molecule, the possibility that RS17 blocks the interaction between mouse CD47 and its ligands may not be excluded.

The CD47‐SIRPα signaling pathway is an important mechanism of immune escape for high CD47‐expressing tumor cells. This pathway represents a good target for the development of antitumor drugs, because inhibiting this pathway suppresses the occurrence and development of tumors. The current study provides a proof of concept that CD47 antagonists can block the CD47 and SIRPα interaction. This promotes tumor cell phagocytosis in vitro and inhibits tumor growth in vivo. Daily subcutaneous injections of 20 mg/kg RS17 resulted in more than a 50% inhibition of tumor growth (Figures 6 and 7), thus, confirming the efficacy of this treatment. Furthermore, the CD47/SIRPα signaling pathway may be used in combination with other signaling inhibitors to produce a synergistic antitumor effect. For example, anti‐CD47 antibodies in combination with Rituximab that targets CD20, showed a greater antitumor effect in non‐Hodgkin's lymphoma compared with either agent alone.^14^ Further study and chemical modifications are warranted to determine the efficacy of RS17 and related peptides for cancer treatment.

## CONFLICTS OF INTEREST

The authors declare no conflict of interest.

## AUTHOR CONTRIBUTIONS

Data curation, Xinmin Wang and Ying Wang; Software, Xinmin Wang; Validation, Jialiang Hu and Hanmei Xu; Writing–original draft, Xinmin Wang; Writing–review & editing, Jialiang Hu and Hanmei Xu.

## ETHICAL APPROVAL

All animal experiments were performed in accordance with the Ethical Guidelines for Animal Health Institutions.

## Supporting information

Fig S1

## Data Availability

The data used to support the findings of this study are available from the corresponding author upon request.

## References

[1] Brown EJ , Frazier WA . Integrin‐associated protein (CD47) and its ligands. Trends Cell Biol. 2001;11(3):130‐135. 11306274 10.1016/s0962-8924(00)01906-1

[2] Reinhold MI , Lindberg FP , Plas D , Reynolds S , Peters MG , Brown. EJ . In vivo expression of alternatively spliced forms of integrin‐associated protein (CD47). J Cell Sci. 1995;108:3419‐3425. 8586654 10.1242/jcs.108.11.3419

[3] Hatherley D , Graham SC , Turner J , Harlos K , Stuart DI , Barclay AN . Paired receptor specificity explained by structures of signal regulatory proteins alone and complexed with CD47. Mol Cell. 2008;31(2):266‐277. 18657508 10.1016/j.molcel.2008.05.026

[4] Sick E , Jeanne A , Schneider C , Dedieu S , Takeda K , Martiny L . CD47 update: a multifaceted actor in the tumour microenvironment of potential therapeutic interest. Br J Pharmacol. 2012;167(7):1415‐1430. 22774848 10.1111/j.1476-5381.2012.02099.x PMC3514757

[5] Isenberg JS , Ridnour LA , Dimitry J , Frazier WA , Wink DA , Roberts DD . CD47 is necessary for inhibition of nitric oxide‐stimulated vascular cell responses by thrombospondin‐1. J Biol Chem. 2006;281(36):26069‐26080. 16835222 10.1074/jbc.M605040200

[6] Barclay AN . Signal regulatory protein alpha (SIRPα)/CD47 interaction and function. Curr Opin Immunol. 2011;21(1):47‐52. 10.1016/j.coi.2009.01.008 PMC3128989 19223164

[7] Mateo V , Lagneaux L , Bron D , et al. CD47 ligation induces caspase‐independent cell death in chronic lymphocytic leukemia. Nat Med. 1999;5(11):1277‐1284. 10545994 10.1038/15233

[8] Chao MP , Majeti R , Weissman IL . Programmed cell removal: a new obstacle in the road to developing cancer. Nat Rev Cancer. 2011;12(1):58‐67. 22158022 10.1038/nrc3171

[9] Okazawa H , Motegi S , Ohyama N , et al. Negative regulation of phagocytosis in macrophages by the CD47‐SHPS‐1 system. J Immunol. 2005;174(4):2004‐2011. 15699129 10.4049/jimmunol.174.4.2004

[10] Neil Barclay A , van den Berg TK . The interaction between signal regulatory protein alpha (SIRPα) and CD47: structure, function, and therapeutic target. Annu Rev Immunol. 2014;32:25‐50. 24215318 10.1146/annurev-immunol-032713-120142

[11] Liu X , Kwon H , Li Z , Fu Y . Is CD47 an innate immune checkpoint for tumor evasion? J Hematol Oncol. 2017;10(1):12. 28077173 10.1186/s13045-016-0381-z PMC5225552

[12] Tsai RK , Discher DE . Inhibition of “self” engulfment through deactivation of myosin‐II at the phagocytic synapse between human cells. J Cell Biol. 2008;180(5):989‐1003. 18332220 10.1083/jcb.200708043 PMC2265407

[13] Sosale NG , Rouhiparkouhi T , Bradshaw AM , Dimova R , Lipowsky R , Discher DE . Cell rigidity and shape override CD47's “self”‐signaling in phagocytosis by hyperactivating myosin‐II. Blood. 2015;125(3):542‐552. 25411427 10.1182/blood-2014-06-585299 PMC4296014

[14] Chao MP , Alizadeh AA , Tang C , et al. Anti‐CD47 antibody synergizes with rituximab to promote phagocytosis and eradicate non‐hodgkin lymphoma. Cell. 2010;142(5):699‐713. 20813259 10.1016/j.cell.2010.07.044 PMC2943345

[15] Zhang H , Lu H , Xiang L , et al. HIF‐1 regulates CD47 expression in breast cancer cells to promote evasion of phagocytosis and maintenance of cancer stem cells. Proc Natl Acad Sci USA. 2015;112(45):E6215‐E6223. 26512116 10.1073/pnas.1520032112 PMC4653179

[16] Chan KS , Espinosa I , Chao M , et al. Identification, molecular characterization, clinical prognosis, and therapeutic targeting of human bladder tumor‐initiating cells. Proc Natl Acad Sci USA. 2009;106(33):14016‐14021. 19666525 10.1073/pnas.0906549106 PMC2720852

[17] Fang X , Chen C , Xia F , et al. CD274 promotes cell cycle entry of leukemia‐initiating cells through JNK/Cyclin D2 signaling. J Hematol Oncol. 2016;9(1):124. 27855694 10.1186/s13045-016-0350-6 PMC5114730

[18] Sick E , Boukhari A , Deramaudt T , et al. Activation of CD47 receptors causes proliferation of human astrocytoma but not normal astrocytes via an Akt‐dependent pathway. Glia. 2011;59(2):308‐319. 21125662 10.1002/glia.21102

[19] Chao MP , Tang C , Pachynski RK , Chin R , Majeti R , Weissman IL . Extranodal dissemination of non‐Hodgkin lymphoma requires CD47 and is inhibited by anti‐CD47 antibody therapy. Blood. 2011;118(18):4890‐4901. 21828138 10.1182/blood-2011-02-338020 PMC3208297

[20] Sikic BI , Lakhani N , Patnaik A , et al. First‐in‐human, first‐in‐class phase I trial of the anti‐CD47 antibody Hu5F9‐G4 in patients with advanced cancers. J Clin Oncol. 2019;37(12):946‐953. 30811285 10.1200/JCO.18.02018 PMC7186585

[21] Ott PA , Hodi FS , Kaufman HL , Wigginton JM , Wolchok JD . Combination immunotherapy: a road map. J Immunother Cancer. 2017;5(1):1‐5. 28239469 10.1186/s40425-017-0218-5 PMC5319100

[22] Lau JL , Dunn MK . Therapeutic peptides: historical perspectives, current development trends, and future directions. Bioorganic Med Chem. 2018;26(10):2700‐2707. 10.1016/j.bmc.2017.06.052 28720325

[23] Padhi A , Sengupta M , Sengupta S , Roehm KH , Sonawane A . Antimicrobial peptides and proteins in mycobacterial therapy: current status and future prospects. Tuberculosis. 2014;94(4):363‐373. 24813349 10.1016/j.tube.2014.03.011

[24] Fosgerau K , Hoffmann T . Peptide therapeutics: current status and future directions. Drug Discov Today. 2015;20:122‐128. 25450771 10.1016/j.drudis.2014.10.003

[25] lmatar M , Makky EA , Yakici G , Var I , Kayar B , Koksal F . Antimicrobial peptides as an alternative to anti‐tuberculosis drugs. Pharmacol Res. 2018;128:288‐305. 29079429 10.1016/j.phrs.2017.10.011

[26] Shimura H , Tanaka R , Shimada Y , Yamashiro K , Hattori N , Urabe T . Glycyl‐alanyl‐histidine protects PC12 cells against hydrogen peroxide toxicity. BMC Biochem. 2017;18(1):14. 29166856 10.1186/s12858-017-0089-x PMC5700669

[27] Joncour VL , Laakkonen P . Seek & destroy, use of targeting peptides for cancer detection and drug delivery. Bioorganic Med Chem. 2018;26(10):2797‐2806. 10.1016/j.bmc.2017.08.052 28893601

[28] Rastogi S , Shukla S , Kalaivani M , Singh GN . Peptide‐based therapeutics: quality specifications, regulatory considerations, and prospects. Drug Discov Today. 2019;24(1):148‐162. 30296551 10.1016/j.drudis.2018.10.002

[29] Boohaker RJ , Sambandam V , Segura I , Miller J , Suto M , Xu B . Rational design and development of a peptide inhibitor for the PD‐1/PD‐L1 interaction. Cancer Lett. 2018;434:11‐21. 29920293 10.1016/j.canlet.2018.04.031

[30] Scognamiglio PL , Natale CD , Perretta G , Marasco D . From peptides to small molecules: an intriguing but intricated way to new drugs. Curr Med Chem. 2013;20(31):3803‐3817. 23895692 10.2174/09298673113209990184

[31] Chang H , Liu B , Qi Y , et al. Blocking of the PD‐1/PD‐L1 Interaction by a D‐peptide antagonist for cancer immunotherapy. Angew Chem Int Ed Engl. 2015;54(40):11760‐11764. 26259671 10.1002/anie.201506225

[32] Geng Q , Jiao P , Jin P , Su G , Dong J , Yan B . PD‐1/PD‐L1 inhibitors for immuno‐oncology: from antibodies to small molecules. Curr Pharm. 2018;23(39):6033‐6041. 10.2174/1381612823666171004120152 28982322

[33] Vlieghe P , Lisowski V , Martinez J , Khrestchatisky M . Synthetic therapeutic peptides: Science and market. Drug Discov Today. 2010;15(1–2):40‐56. 19879957 10.1016/j.drudis.2009.10.009

[34] Miller KD , Nogueira L , Mariotto AB , et al. Cancer treatment and survivorship statistics, 2019. CA Cancer J Clin. 2019;69(5):363‐385. 31184787 10.3322/caac.21565

[35] Ma WW , Adjei AA . Novel agents on the horizon for cancer therapy. CA Cancer J Clin. 2009;59(2):111‐137. 19278961 10.3322/caac.20003

[36] Wang SD , Li HY , Li BH , et al. The role of CTLA‐4 and PD‐1 in anti‐tumor immune response and their potential efficacy against osteosarcoma. Int Immunopharmacol. 2016;38:81‐89. 27258185 10.1016/j.intimp.2016.05.016

[37] Palomo JM . Solid‐phase peptide synthesis: An overview focused on the preparation of biologically relevant peptides. RSC Adv. 2014;4:32658‐32672.

[38] Yassin S , Hu J , Xu H , Li C , Setrerrahmane S . In vitro and in vivo activities of an antitumor peptide HM‐3: a special dose‐efficacy relationship on an HCT‐116 xenograft model in nude mice. Oncol Rep. 2020;43(4):1349. 32323854 10.3892/or.2020.7503

[39] Jerabek‐willemsen M , Andre T , Wanner R , et al. MicroScale thermophoresis: interaction analysis and beyond. J Mol Struct. 2014;1077:101‐113.

[40] Kuwada K , Kagawa S , Yoshida R , et al. The epithelial‐to‐mesenchymal transition induced by tumor‐associated macrophages confers chemoresistance in peritoneally disseminated pancreatic cancer. J Exp Clin Cancer Res. 2018;37(1):307. 30537992 10.1186/s13046-018-0981-2 PMC6288926

[41] Ji G , Chen R , Zheng J . Atractylenolide I inhibits lipopolysaccharide‐induced inflammatory responses via mitogen‐activated protein kinase pathways in RAW264.7 cells. Immunopharmacol Immunotoxicol. 2014;36(6):420‐425. 25270720 10.3109/08923973.2014.968256

[42] Majeti R , Chao MP , Alizadeh AA , et al. CD47 is an adverse prognostic factor and therapeutic antibody target on human acute myeloid leukemia stem cells. Cell. 2009;138(2):286‐299. 19632179 10.1016/j.cell.2009.05.045 PMC2726837

