# Supplementary material for: An antitumor peptide RS17‐targeted CD47, design, synthesis, and antitumor activity
Source: Cancer Med. 2021 Feb 24;10(6):2125–36. doi: 10.1002/cam4.3768 (PMC7957188; doi:10.1002/cam4.3768)
Supplement: Supplementary file 1 — Fig S1 [file CAM4-10-2125-s001.pdf]

A

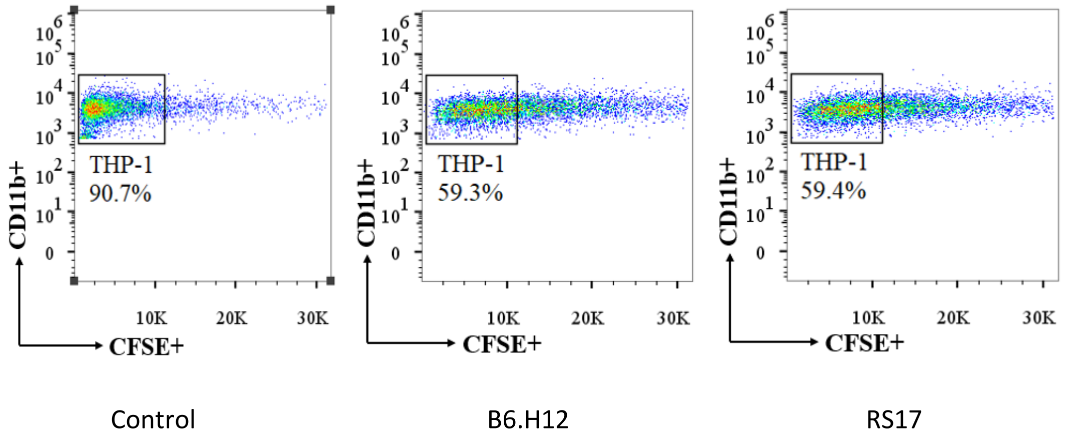

B

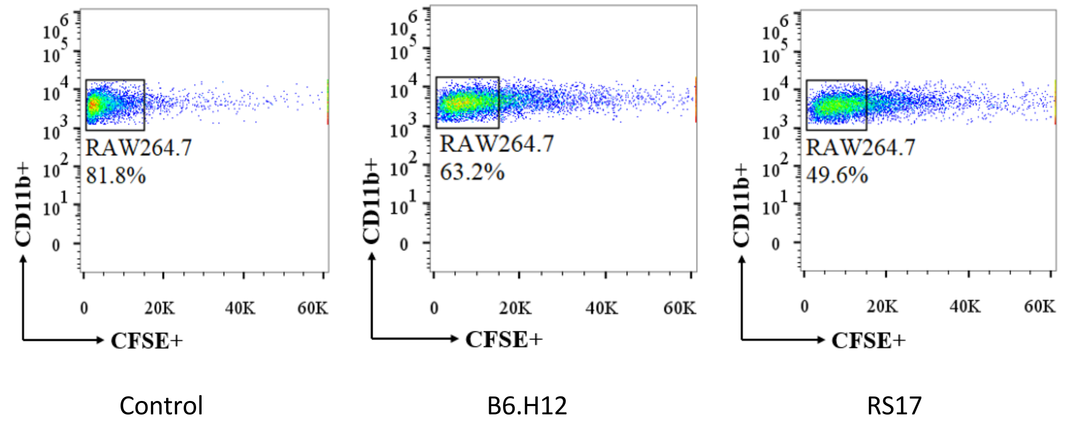

sFigure 1. Phagocytosis was detected and quantitated by flow cytometry. Macrophages were labeled with APC-Anti-CD11b antibodies. Increased phagocytosis was evident by an increase in green fluorescence (CFSE) of the macrophages. (A) Quantitation of HepG2 cell phagocytosis by THP-1 cells using flow cytometry. (B) Quantitation of HepG2 cell phagocytosis by RAW264.7 cells using flow cytometry.
